# The Influence of Gender and Sexual Orientation on Alcohol Use and Alcohol-Related Problems: Toward a Global Perspective

**DOI:** 10.35946/arcr.v38.1.15

**Published:** 2016

**Authors:** Tonda L. Hughes, Sharon C. Wilsnack, Lori Wolfgang Kantor

**Affiliations:** Tonda L. Hughes, Ph.D., R.N., F.A.A.N., is professor and associate dean for global health and co-director, Building Interdisciplinary Research Careers in Women’s Health (BIRCWH), University of Illinois at Chicago, Chicago, Illinois. Sharon C. Wilsnack, Ph.D., is the Chester Fritz Distinguished Professor, Department of Psychiatry and Behavioral Science, University of North Dakota School of Medicine & Health Sciences, Grand Forks, North Dakota. Lori Wolfgang Kantor, M.A., is a science editor at *Alcohol Research: Current Reviews.*

**Keywords:** Alcohol consumption, alcohol use patterns, heavy drinking, alcohol- related problems, gender, sexual orientation, sexual minority, heterosexual, men, women, global perspective, literature review

## Abstract

Although there are wide differences in alcohol use patterns among countries, men are consistently more likely than women to be drinkers and to drink heavily. Studies of alcohol use among sexual minorities (SMs), however, reflect a more complex picture. Such research has found higher rates of alcohol use and alcohol-related problems among SM persons than among heterosexuals and greater differences between SM and heterosexual women than between SM and heterosexual men. A variety of factors may contribute to differences in alcohol use and alcohol-related problems between men and women and between SM and heterosexual people. An improved understanding of these factors is important to guide prevention and treatment efforts. Although there is a dearth of literature on use of alcohol by SMs in many parts of the world, especially lower- and middle-income countries, we attempt to review and integrate the sparse data that are available from these lower-resourced countries. The global perspective presented in this article is the first attempt to go beyond a general review of literature in the Western world to document the gender paradox in alcohol use among heterosexuals and SMs in diverse countries worldwide.

The prevalence of alcohol use and the contrast between the drinking patterns of men and women vary widely across the globe. For instance, rates of current drinking ranged from 3 percent and 37 percent for women and men, respectively, in the Indian state of Karnataka to 94 percent and 97 percent for women and men in Denmark (Wilsnack et al. 2009). Overall, however, men have higher rates of alcohol use than women, both in the United States (Substance Abuse and Mental Health Services Administration [SAMHSA] 2013) and globally. In a multinational study of 35 countries (Gender, Alcohol, and Culture: An International Study [GENACIS]), Wilsnack and colleagues (2009) found that men were consistently more likely than women to be current drinkers and to engage in high-volume drinking, high-frequency drinking (5 or more days per week), and heavy episodic drinking. Women were more likely to be lifetime nondrinkers and to be former drinkers.

These patterns are quite different among sexual-minority women (SMW) and sexual-minority men (SMM). Although many large-scale surveys of alcohol and other drug (AOD) use have not included questions about sexual orientation, those that do show smaller gender differences in alcohol use and related problems among SMs than among heterosexuals. Notably, sexual-orientation–related disparities in AOD use are larger for women than for men. That is, SMW differ more in their rates of AOD use and related problems from heterosexual women than SMM differ from heterosexual men (Drabble et al. 2005; McCabe et al. 2009; Talley et al. 2014). This article examines the relationships that gender and sexual orientation have to alcohol use and alcohol-related problems, using available literature in the United States and globally, and reviews some of the factors that seem to influence these relationships.

## Sex versus Gender Differences in Alcohol Use and Related Problems

Sex differences refer to biological characteristics such as anatomy and physiology that distinguish female and male bodies. For example, differences in body composition partly explain why women consistently drink less than men. Because women’s bodies generally contain less water than men’s bodies, alcohol becomes less diluted, and women therefore reach higher blood alcohol levels than men even if both drink the same amount (Holmila and Raitasalo 2005).

Gender influences refer to the socially constructed roles, responsibilities, attitudes, behavioral norms, and relative power that a society differentially attributes to women and men. Research shows that countries or cultures with the largest differences in gender roles also have the largest differences between men’s and women’s drinking (Wilsnack et al. 2000). Therefore, social and cultural factors must be considered when attempting to understand gender differences in alcohol use across countries.

### Gender Roles and Alcohol Use

Differences in men’s and women’s alcohol use often reflect gender roles and cultural expectations. Men may use drinking to demonstrate masculinity, facilitate aggression, exert power, and take risks. For these reasons, men may have greater motivation to drink than women. For example, research shows that risk taking is associated with heavy drinking among men but that women are more likely than men to use risk- reduction strategies when drinking (Iwamoto et al. 2011; Nguyen et al. 2011). In addition, a culture’s acceptance of public drinking and intoxication for men but not women can serve to reinforce male superiority over women in status and authority in that culture. Whereas men have used drinking as a way to excuse themselves from responsibilities at work or home, women’s drinking has traditionally been limited by their roles as mothers and caretakers and by the belief that drinking may have a more detrimental effect on their social behavior and their ability to fulfill responsibilities and to control their sexuality (Kuntsche et al. 2009, 2011). Women also are often expected to rein in the drinking of their male partners (Holmila and Raitasalo 2005).

Women who drink are more likely than men to stop drinking. This may be related to their generally lower levels of drinking, their social roles, and the fact that some women do not resume drinking (or return to pre-pregnancy levels) after pregnancy. However, a review of research examining birth cohorts and alcohol use across countries found high rates of heavy episodic drinking among women in younger cohorts in North America and Europe, suggesting a narrowing of the gender gap and a potential shift in social attitudes regarding gender and alcohol use (Keyes et al. 2011). In Finland, an examination of survey data collected over a period of 40 years suggests a cultural shift toward greater alcohol use, especially by women. Weekly drinking, frequency of moderate drinking, quantity of alcohol consumed per occasion, and intoxication increased among both genders over time but proportionately more among women. Drinking at home increased more than drinking in bars, and home drinking increasingly occurred only in the company of partners (Mäkelä et al. 2012). An analysis of survey data from Hispanics living in major U.S. cities found that high acculturation was associated with a higher volume of drinking and greater likelihood of binge drinking among women but not men (Vaeth et al. 2012), perhaps reflecting the greater tolerance of women’s drinking in the United States.

Employment and other social roles are believed to be protective against drinking problems among heterosexual men and women. Jobs and social responsibilities tend to promote enhanced self-esteem and offer greater social support, and they entail responsibilities and more intensive social monitoring that may discourage excessive drinking. However, in part because of societal stigma and discrimination, fewer lesbian women and gay men engage in traditional roles such as marriage, childbearing, and childrearing or have responsibilities associated with social roles believed to limit alcohol use (especially among women) in the general population (Hughes 2005). Even SM couples in long-term relationships find less support for their relationships than do unmarried heterosexual cohabiting couples. For SM couples who do have children, the stressors associated with parenting may be exacerbated. For example, many lesbian and gay parents must deal with the realistic fear of custody battles over competency to raise children, homophobic remarks made to their children, and disclosing their sexual orientation to the children and others.

Efforts to reduce alcohol misuse and related problems among women and men (both heterosexual and sexual minority) should take into account cultural expectations regarding gender roles and alcohol use, as well as contemporary social and cultural changes that may be responsible for a narrowing gap between men’s and women’s drinking in some cultures.

### Gender Differences in Alcohol Use Among Sexual Minorities

McCabe and colleagues (2009) analyzed data from the National Epidemiologic Survey on Alcohol and Related Conditions (NESARC), a nationally representative survey of U.S. adults. They reported that, among those who identified themselves as SM based on sexual identity, behavior, or attraction, lesbian women had more than 3 times greater odds of lifetime alcohol use disorders and of any lifetime substance use disorder than did heterosexual women. In contrast, the odds of lifetime alcohol use disorders for men with histories of only male sex partners were significantly lower than those for men who reported only female sex partners. Similarly, in a study based on data from the 2000 National Alcohol Survey, Drabble and colleagues (2005) reported that, among current drinkers, lesbians were approximately 7 times more likely and bisexual women nearly 6.5 times more likely than heterosexual women to meet *Diagnostic and Statistical Manual, 4th Edition* (American Psychiatric Association 1994) criteria for alcohol dependence. Lesbians were approximately 11 times more likely and bisexual women 8 times more likely to report 2 or more negative social consequences related to drinking compared with heterosexual women. Seeking treatment or other types of help for an alcohol problem was 8 times more likely among lesbians and 4 times more likely among bisexual women than among heterosexual women. There were no significant differences between SM and heterosexual men on any of these outcomes.

This gender-related pattern is similar among youth. In an analysis of data from the Youth Risk Behavior Surveillance System (YRBSS) survey, Talley and colleagues (2014) found that, among 13- to 18-year-olds surveyed, differences in alcohol use outcomes were greater between SM and heterosexual girls than between SM and heterosexual boys. Notably, SM girls reported higher rates of lifetime alcohol use and past-month heavy episodic drinking than did SM boys, heterosexual girls, or heterosexual boys. For instance, 30 percent of SM girls reported past-month heavy episodic drinking compared with 25.4 percent of SM boys, 16.4 percent of heterosexual girls, and 19.3 percent of heterosexual boys.

Studies of alcohol use among SMs outside the United States generally show smaller differences between SM and heterosexual populations, especially for men. For example, in a study examining sexual orientation differences in health risk behaviors among 1,725 15- to 21-year-old vocational school students in northern Thailand, van Griensven and colleagues (2004) found that AOD use patterns among SM females were similar to those of heterosexual males, whereas patterns of SM males were similar to those of heterosexual females. The authors speculate that one explanation for this pattern may be that SM males tend to socialize with heterosexual females who are less likely to use AODs and therefore are less likely to use substances themselves.

Using data from the GENACIS project, Bloomfield and colleagues (2011) analyzed alcohol use information from general-population surveys from 14 countries in Europe, Latin America, and North America. The researchers examined high-volume drinking (average daily consumption greater than 20 g of ethanol [pure alcohol] for women and greater than 30 g for men) and heavy single-occasion drinking (at least monthly consumption of large quantities of alcohol [in most countries, 60 g or more of ethanol in a day]) among heterosexual and SM respondents (defined on the basis of gender of romantic or cohabiting partner). In North America, SMW were significantly more likely than heterosexual women to report high-volume drinking and heavy single- occasion drinking, but no differences were found among men on these outcomes.^1^ In the European countries, high-volume drinking was similar for SM and heterosexual women, and both drinking outcomes were similar for SM and heterosexual men.^2^ Findings from the other regions examined either showed no significant differences between SM and heterosexual respondents or too few cases of high-volume or heavy single-occasion drinking to make comparisons.

In a meta-analysis of 25 studies from 8 countries in Europe, North America, Australia, and New Zealand, King and colleagues (2008) concluded that the risk of past-year AOD dependence was 50 percent higher among gay men, lesbian women, and bisexual men and women than among heterosexual men and women, with lesbian and bisexual women at especially high risk.

Nonadherence to traditional gender roles for women may influence drinking among SMW—especially in lower- and middle-income countries where the value placed on traditional gender roles remains strong. Using data from the 2005 National Youth Survey, a nationally representative sample of 12- to 29-year-olds in Mexico, Ortiz-Hernandez and colleagues (2009) found higher prevalence of alcohol use among lesbian and bisexual females, but not among gay and bisexual males, than among their heterosexual counterparts. The authors concluded that results support findings from previous studies of greater differences in the relationship between sexual orientation and alcohol use among women than among men. They further suggest that higher frequency and volume of drinking among SMW may be related to increased socialization in bars and more widespread adoption of masculine traits compared with heterosexual women. These findings are consistent with those from a study conducted in Taiwan, where the authors (Kuang et al. 2004) found adoption of nontraditional gender roles and higher rates of drinking among SMW than among heterosexual women.

### Age Differences in Drinking

Rates of drinking generally decline with age for both men and women (World Health Organization 2014), although research with older adults suggests that men reduce their drinking later than women do (Brennan et al. 2011). In 2012, the proportion of people in the United States reporting at least 1 drink in the previous 30 days (i.e., current drinkers) decreased from 69.2 percent among 21- to 25-year-olds to 60.1 percent among 40- to 44-year-olds and 53.1 percent among 60- to 64-year-olds (SAMHSA 2013). The same survey also found that 61.2 percent of men ages 26 and older were current drinkers, compared with 50.4 percent of women in the same age range. International surveys, however, show a somewhat different pattern. Based on GENACIS data, Wilsnack and colleagues (2009) reported that the prevalence of current drinking declined consistently with age in only a minority of the surveys for which 3 age groups were available. The prevalence of high-volume drinking declined with age among men in only 3 of the 34 surveys, and among women in only 11 of the 34 surveys. Most age-related declines in high-volume drinking occurred in high-income countries: Europe, the United States, Australia, and New Zealand.

Alcohol use among SM groups also decreases with age, but the declines tend to be smaller and to occur at later ages relative to heterosexuals. For example, in a community-based study of 447 women who identified as lesbian or bisexual, Hughes and colleagues (2006) found that, in contrast with the tendency for drinking among women in the general population to decline with age, there was relatively little variation in drinking rates among SMW across 4 age groups (≤30 years, 31–40 years, 41–50 years, >50 years). Using data from the 2003–2010 Washington State Behavioral Risk Factor Surveillance surveys, Fredriksen-Goldsen and colleagues (2013) found that lesbian and bisexual women ages 50 or older were significantly more likely than their age-matched heterosexual counterparts (adjusted odds ratio [AOR] = 1.43) to drink excessively, as were older (50 years or older) gay and bisexual men compared with older heterosexual men (AOR = 1.47). In an earlier study, McKirnan and Peterson (1989*a*) found similar rates of alcohol problems among 18- to 25-year-old gay men (26 percent) and heterosexual men (29 percent), but higher rates among gay men (19 percent) than heterosexual men (7 percent) who were ages 41–60. In the same study, lesbian women in the oldest age group (age 41–60) were 3 times as likely to report alcohol-related problems as were heterosexual women in that age group (15 percent vs. 4.5 percent).

### Race/Ethnicity Differences in Drinking

Research examining alcohol-related problems across racial/ethnic groups in the United States suggests that gender and sexual orientation are important factors in this relationship. A recent analysis using pooled data from the 2005 and 2010 U.S. National Alcohol Surveys examined heavy drinking and alcohol-related consequences for White, Black, and Hispanic men and women (Witbrodt et al. 2014). The study found that, across all levels of heavy drinking, Black women drinkers had greater odds of alcohol dependence relative to White women drinkers, but no other significant differences were noted among the 3 groups of women.^3^ Women showed low rates of alcohol dependence and alcohol-related consequences across ethnicities, except that Hispanic women were marginally more likely than White women to experience arguments and fights resulting from their drinking. Racial/ethnic differences were greater among men. Black men with no/low levels of heavy drinking had significantly greater odds than White men of having 3 or more alcohol-dependence symptoms and of having 2 or more negative drinking consequences. Compared with White men, Hispanic men who reported low or moderate heavy drinking also had significantly elevated odds of alcohol dependence. The authors suggest that the gender disparity may be partly explained by social norms that limit women’s drinking across racial/ethnic boundaries.

Among SMs, there seem to be different associations among race/ethnicity, gender, and drinking. SMW who belong to racial/ethnic minorities seem to be at greater risk for AOD problems than heterosexual non-White women, whereas SM non-White men seem to be at comparable or less risk than heterosexual non-White men (Cochran et al. 2007*b*; Kim and Fredriksen-Goldsen 2012). In a race- and ethnicity-diverse community sample of SMW, Hughes and colleagues (2006) found that Black respondents were nearly four times more likely than White respondents to report heavy drinking. Mereish and Bradford (2014) found that Black and Hispanic SMW were more likely than Black and Hispanic heterosexual women and White SMW to report having had an alcohol- or other drug-use problem. Black and Hispanic SMM, however, did not differ in their risk compared with Black and Hispanic heterosexual men, and they had lower risk than White SMM.

Both White and non-White SM youth are at risk for alcohol problems. Talley and colleagues (2014) reported that, among 13- to 18-year-olds, White SMs were more likely than White heterosexuals to report ever drinking (79.9 percent vs. 69.1 percent), and Asian SMs were more likely than their heterosexual counterparts to report drinking (54.8 percent vs. 46.2 percent). Although bisexual White and racial/ethnic minorities initiated drinking at similar ages, heterosexual racial/ethnic minorities were significantly younger than their White counterparts when they had their first drink. For young women, there were fewer racial/ethnic differences in drinking among SMs than among heterosexual women.

### Socioeconomic Status and Drinking

In the general population, higher levels of socioeconomic status (SES) are associated with more frequent alcohol use, whereas lower SES often is associated with heavier drinking (Huckle et al. 2010), although these patterns vary somewhat across cultures (Bloomfield and Mäkelä 2010; Bloomfield et al. 2006). With regard to gender, analyses of survey data from the Netherlands showed that abstinence was inversely associated with educational level for both men and women. Among male drinkers, excessive drinking and very excessive drinking were more prevalent in the group with the lowest educational level. There was no significant relationship between educational level and prevalence of excessive drinking among women (van Oers et al. 1999).

Studies of adolescent alcohol use and SES in England (Melotti et al. 2013) and Brazil (Locatelli et al. 2012) suggest greater risk for higher-SES young people. In England, higher household income was associated with greater risk of alcohol use and problem use, especially among girls (Melotti et al. 2013). A study that compared alcohol use among Slovak adolescents in 1998 and 2006 found no socioeconomic differences among boys and greater likelihood for girls of high SES to be drinkers in 1998, but not in 2006 (Pitel et al. 2013).

Although scant research has examined the relationship between SES and alcohol use among SMs, studies of education and income are relevant. Some research has found that same-sex couples who live together earn less than heterosexual married couples, possibly because of workforce discrimination (Badgett and Lee 2001), whereas other studies find that cohabiting same-sex couples have more advantages in terms of education and income than opposite- sex cohabiting couples (Gates 2012, 2013; Kastanis and Wilson 2014; Krivickas 2010). In contrast, bisexual adults often show greater disadvantage in earnings than gay, lesbian, and heterosexual adults (Gates 2012). In terms of general health, same-sex cohabitors report poorer health than their heterosexual married counterparts at the same SES levels (Liu et al. 2013). In the only study we located that examined the relationship between educational level and substance use disorders (and other mental health problems) among SMs, Barnes and colleagues (2014) found that sexual- orientation disparities in substance use disorder rates were smaller among respondents with bachelor’s degrees than among those with less education. These data were from the NESARC.

In addition to education and income, marital and parental status are likely associated with risk of heavy or problematic drinking. For example, in a nationally representative study of Australian women ages 25–30, Hughes and colleagues (2010*b*) found that, compared with married women, those in relationship categories more common among SMW (e.g., de facto, never married) reported significantly higher odds of AOD use. In addition, lower levels of education and not having children were each associated with significantly higher odds of at-risk drinking.

Using data from the U.S. National Health Interview Study, Denney and colleagues (2013) also found that same-sex cohabiting couples had both higher household incomes and higher educational levels than opposite-sex married couples and cohabiting couples. However, after adjusting for socioeconomic differences, same-sex cohabiting couples had worse health than opposite-sex married couples and similar health as opposite-sex cohabiting couples. These researchers also found a significant protective effect of having children in the household on partnered men’s and women’s self- assessed health (heterosexual and SMs alike), but the effect was significantly greater for heterosexual married women.

## Factors Associated With Alcohol Use Among Sexual Minorities

### Minority Stress

A variety of potential risk factors have been suggested to explain the higher prevalence of alcohol use and alcohol- related problems among SMs. The predominant theoretical explanation is minority stress (Meyer 2003). Underlying this perspective are the assumptions that minority stressors are unique (not experienced by nonstigmatized populations), chronic (related to social and cultural structures), and socially based (stemming from social processes, institutions, and structures). The minority stress perspective describes stress processes that include experiences of prejudice, expectations of such prejudice and of rejection (stigma consciousness), hiding, concealing, internalized homophobia, and ameliorative coping processes. Expectations of prejudice and discrimination and the vigilance that such expectations require vary based on individual and environmental contexts, but all SM persons are assumed to internalize society’s negative attitudes toward homosexuality to some degree (internalized homophobia) (Meyer 2003).

In a large study using quantitative and qualitative methods to examine mental health and well-being among SMs in Ireland, more than 40 percent of 1,100 survey respondents reported that their drinking made them “feel bad or guilty,” and almost 60 percent indicated feeling that they should reduce their alcohol consumption. Qualitative findings strongly suggested that self-medication to cope with minority stress was a primary motive for regular or heavy alcohol consumption (Mayock et al. 2008).

Analyses of the National Survey on Midlife Development in the United States found that compared with heterosexuals, SM women and men more frequently reported both discrete discrimination events (e.g., being fired from a job) and day-to-day discrimination (e.g., being called names or insulted) (Mays and Cochran 2001). Perceived discrimination was associated with reduced quality of life and with indicators of psychiatric morbidity in both SM and heterosexual respondents. Other studies have shown that harassment and discrimination based on sexual orientation are associated with psychological distress (Herek et al. 1997; Lewis et al. 2001, 2003; Meyer 1995), loneliness (Szymanski and Chung 2001), and lower self- esteem (Szymanski et al. 2001). Relatively few studies have examined the impact of such stressors on the drinking behaviors of SMs (Hatzenbuehler et al. 2008, 2010; McCabe et al. 2010). In an early study of lesbian women and gay men, McKirnan and Peterson (1989*b*) found that stress was associated with alcohol- or drug-related problems in high-vulnerability gay men (those with greater orientation to gay bars and positive expectancies about the tension-reducing effects of alcohol). However, such associations were not statistically significant for lesbians or for low-vulnerability gay men.

### Drinking Norms

Drinking behavior is governed to a large extent by social structures (rules, role expectations, norms, and values) of the individual’s cultural group and by the drinking behavior of peers. Because of their history of being excluded and discriminated against in mainstream settings, many SM people have traditionally found bars to be an important venue for social interaction. Findings from the 2000 National Alcohol Survey conducted in the United States (Trocki et al. 2005) indicated that SMW spend more time in bars and party settings and consume more alcohol in these settings than do hetero- sexual women. Although gay men spent more time in bars than did bisexual or heterosexual men, rates of heavy drinking among men did not vary by sexual orientation across settings.

According to Cochran and colleagues (2012), the adoption of a minority sexual identity and affiliation with gay-identified communities increase exposure to more tolerant social norms regarding AOD use. These researchers found that SMs report more tolerant norms about AOD use and greater availability of these substances. These two factors also mediated a substantial portion of the relationship between minority sexual orientation and substance use.

### Experiences of Victimization

Abuse, violence, and victimization are considered major life stressors and are consistently linked with long-term adverse consequences, including hazardous drinking and alcohol use disorder (Briere 1988; Dube et al. 2002; Kendler et al. 2000; Nelson et al. 2002; Wilsnack et al. 2004). For example, a review of research linking childhood abuse to alcohol use and related problems in adulthood has estimated that globally, a history of child sexual abuse accounts for 4 percent to 5 percent of alcohol misuse/dependence in men and 7 percent to 8 percent in women (Andrews et al. 2004).

SMs are at increased risk for childhood abuse compared with hetero- sexuals (Alvy et al. 2013; Austin et al. 2008; Drabble et al. 2013; Hughes et al. 2010*a*, 2014; Tjaden et al. 1999), thereby further increasing their risk of developing alcohol-related problems. Using a pooled sample from two large studies of U.S. women, Wilsnack and colleagues (2008) found that those who identified as lesbian, bisexual, or mostly heterosexual reported significantly higher rates of childhood sexual abuse (CSA) compared with women who identified as exclusively heterosexual. In addition, SMW reported significantly higher rates of heavy drinking, heavy episodic drinking, and symptoms of potential alcohol dependence than exclusively heterosexual women.

In addition to high rates of CSA, accumulating evidence suggests that many other forms of lifetime sexual and physical abuse, violence, and victimization also are more common among SMs (Balsam et al. 2005; Drabble et al. 2013; Hughes et al. 2010*a*). Using the pooled sample described above, Hughes and colleagues (2014) found that SMW were significantly more likely than exclusively heterosexual women to report each of six types of lifetime victimization: CSA, childhood physical abuse, childhood neglect, adult sexual assault, adult physical assault, and intimate- partner violence. The number of types of victimization experiences was positively associated with hazardous drinking among both SM and heterosexual women but contributed to higher levels of hazardous drinking among SMW.

Hughes and colleagues (2010*a*) analyzed data from the NESARC. Results supported findings from previous studies suggesting that SM women and men are at higher risk for victimization than their heterosexual counterparts. Lesbian and bisexual women were more than twice as likely as heterosexual women to report any lifetime victimization. Lesbians, gay men, and bisexual women also reported a greater number of victimization experiences. The largest difference between lesbian and heterosexual women was in reports of CSA: 3 times as many lesbians (34.7 percent) as heterosexual women (10.3 percent) reported this experience (see figure). Bisexual women also were more likely than heterosexual women to report CSA, as well as three other lifetime victimization experiences. Women who reported two or more victimization experiences had two to four times the odds of alcohol dependence and drug use disorders as women who reported no victimization. Lesbians who reported childhood neglect had more than 30 times the odds of alcohol dependence as heterosexual women who reported neglect. In contrast, although gay men were significantly more likely than heterosexual men to report four of seven victimization experiences, these differences did not increase gay men’s risk of substance use disorders (SUDs). Bisexual men were similar to heterosexual men in prevalence of victimization experiences, but associations between victimization and SUDs were stronger in bisexual men.

In addition to SMW’s higher rates of childhood victimization, the severity of victimization experiences also may vary by sexual orientation. Two recent studies have found that women who self-identify as lesbian report significantly greater severity of CSA (Wilsnack et al. 2012) and of childhood physical abuse (Alvy et al. 2013) than do women who identify as heterosexual.

Higher rates of victimization among SMs, especially SM youth, may be related to gender-atypical appearance and behavior. For example, in a recent review of findings from 12 countries (Australia, Austria, Belgium, Canada, Israel, Japan, the Netherlands, New Zealand, Norway, South Africa, the United Kingdom, and the United States), Collier and colleagues (2013) found that sexual orientation and gender expression were associated with peer victimization, which in turn was related to AOD abuse. Similarly, gender- atypical behavior was associated with more negative parental relationships (D’Augelli et al. 2008; Ryan et al. 2009), a factor that can lead youth to run away from home and/or to be more likely to participate in situations that put them at risk for victimization.

### Societal Attitudes and Policies Regarding SMs

SMs and their families now are experiencing increasing public support and access to legal rights, such as marriage, in some parts of the world. According to the Pew Research Center, as of June 26, 2015, 22 countries worldwide permitted lesbian women and gay men to marry their same-sex partners, and same-sex marriage is legal in some parts of Mexico (Pew Research Center 2015). Although attitudes toward SMs also are changing in some other parts of the world, most people (and thus the majority of SM people) live in countries with strong anti-gay policies. In 2014, it was estimated that 2.79 billion people live in countries where being openly gay or lesbian is punishable by imprisonment or death—a number 7 times greater than those who live in countries with laws that recognize same-sex marriage (Ball 2014).

Increasing evidence throughout many parts of the world documents the negative effects of stigma, discrimination, and criminalization on SM people’s health, including minority stress, depression, and fear of seeking help (Kates 2014). Whether and how the World Health Organization (WHO) should address SM health has been debated over the past few years. Although opposition from a number of African and Middle Eastern countries has prevented this topic from being included on the WHO agenda (Daulaire 2014), the Pan-American Health Organization (PAHO), the WHO regional arm representing the Americas, unanimously passed a resolution addressing SM health, including discrimination in the health sector. This marks the first time any United Nations body has adopted a resolution specifically addressing these issues (PAHO 2012, 2013).

Research suggests that societal norms and policies that discriminate against SMs increase the risk of alcohol use disorder for SMs. For example, one U.S. study that examined the relationship between State-level policies and psychiatric morbidity found that lesbians, gays, and bisexuals who lived in States without protective policies toward SMs (e.g., laws against hate crimes and employment discrimination) had higher odds of alcohol use disorder than those who lived in States with protective policies (Hatzenbuehler et al. 2009). The authors also examined psychiatric morbidity among SMs before (2001–2002) and after (2004–2005) States had enacted same-sex marriage bans (Hatzenbuehler et al. 2010). Mood disorder (36.6 percent), generalized anxiety disorder (248.2 percent), and alcohol use disorder (41.9 percent) all increased significantly among SM residents in these States between the 2 data collection points. Psychiatric disorders did not significantly differ over time among SMs living in States without marriage bans. In addition, the researchers found statistically significant increases in generalized anxiety, panic, and alcohol use disorder among heterosexuals living in States with the bans, but these increases were not of the same magnitude as those experienced by SMs.

## Conclusions/Recommendations/Future Directions

### What Explains the “Gender Paradox”?

This review has documented clear differences in gender-related patterns of alcohol use between heterosexual and SM persons. Specifically, most studies that ask about sexual orientation find that SMW substantially exceed heterosexual women in high-risk drinking and adverse drinking consequences, whereas SMM may exceed heterosexual men but by a much smaller margin, if at all. This creates a “gender paradox”: heterosexual men typically drink much more than heterosexual women, but the reverse is true among SM men and women.

An intriguing question is why these sexual orientation differences exist, and what they can tell us about gender and alcohol use more generally. In our opinion, one important factor contributing to the gender paradox is the differential adoption of traditional gender roles by SMs compared with heterosexuals. There is ample evidence that culturally defined gender roles in most societies link alcohol use (and especially heavier use) more closely with traditional masculine roles than with traditional feminine ones. As discussed earlier, men in many cultures use alcohol to demonstrate masculine gender superiority and power, whereas women’s drinking is limited by cultural beliefs that drinking could threaten their performance of traditional feminine roles as mothers, caretakers, and controllers of men’s drinking (Holmila and Raitasalo 2005; Wilsnack et al. 2005). To the extent that SM persons of both genders reject these traditional gender roles and expectations (Lippa 2000), SMW would be expected to drink more than heterosexual women and SMM would feel less pressure to engage in traditionally masculine heavy drinking. Thus, whereas minority stress may contribute to greater risk of drinking in both SM women and men (Hatzenbuehler 2009; Meyer 2003), relative freedom from traditional gender roles would predict larger increases in drinking by SM women than SM men, reversing the heterosexual pattern of men’s drinking exceeding women’s.

Additional influences may contribute to the gender paradox. For example, gay men may drink less due to weight and body image concerns (Kimmel and Mahalik 2005) or to greater socialization with heterosexual women, who evoke less pressure toward heavy drinking (van Griensven et al. 2004), and SMW’s greater dependence on gay bars as venues for socialization may increase their risks of frequent and/or heavy drinking (Kuang et al. 2004; Trocki et al. 2005). However, the important links between traditional gender roles and heavier versus lighter drinking seem of central importance in understanding both the heavier drinking by heterosexual men than heterosexual women *and* the reversal of this pattern among SM women and SM men. This interpretation of the gender paradox also suggests that social change (and intentional intervention efforts) that produce less gender-role differentiation and greater gender-role flexibility could help to reduce gender-role–related alcohol use and alcohol problems among both heterosexual and SM women and men.

## Research

### Sexual Minority Research

Until the advent of HIV/AIDS in the 1980s, there was almost no funding for SM health research. Since then, apart from HIV/AIDS, there has been relatively little funding for research with SMs—even in the United States, where most of this research has been done. Recently, Coulter and colleagues (2014) conducted a review of grants funded by the National Institutes of Health (NIH). Between 1989 and 2011, apart from studies of HIV/AIDS, only 0.1 percent of all NIH- funded studies focused on SMs. Of these, most have focused on SMM, with only 13.5 percent focusing on SMW and only 13 percent of funded SM studies focusing on alcohol use. The dearth of funding is a major contributor to gaps in knowledge, especially in non-Western countries. In addition, researchers throughout the world who study SM health must move beyond the focus on disease and deviance, to also study strengths and resilience factors among SMs. And just as women (or men) should not be considered a single homogeneous group, SM people are extremely diverse in terms of their health behaviors and health outcomes (Boehmer 2002). Future research must take into account the nuances of gender and gender identity, sexual orientation, and culture as well as economic and social resources.

### Gender and Alcohol Research

To some extent, research on sexual orientation disparities in alcohol use and related problems is following a trajectory similar to that of research on women and alcohol. Until the 1970s, research on alcohol use and misuse gave little attention to drinking by women; when women were even considered, it was assumed that their drinking and its consequences would be similar to those of men. In 1970, only 28 English-language alcohol research articles could be found that included women as research participants (Sandmaier 1980). Research on women’s drinking, and on how gender is related to alcohol use and its consequences, has increased dramatically since the 1970s, to the point where more than 1,000 new articles related to gender and alcohol are published each year (Wilsnack and Wilsnack 2013). Reasons for the increased attention paid to women and gender include effects of the U.S. women’s movement of the 1960s and 1970s, growing awareness of fetal alcohol syndrome and other adverse outcomes of alcohol use in pregnancy, and a gradual recognition in medical and behavioral science that many diseases and disorders could not be understood and adequately prevented or treated without taking into account the multiple ways they are affected by gender.

Like research on SMs, research on women’s drinking initially focused on comparisons between women (as a homogeneous group) and men (as an equally homogeneous group). Only gradually did investigators begin to explore variations *within* gender groups—by age, race/ethnicity, and socioeconomic status, and eventually by sexual orientation. We hope that this trend toward greater attention to within-group variations will also continue in research on SMs, and that the sections on demographic differences in this article (e.g., by age, race/ethnicity, and SES) will help to accelerate this trend.

### Prevention, Intervention, and Treatment

Research on treatment for AOD use disorders among women and men in the general population comprises a large and growing body of literature whose review is beyond the scope of this article. However, it may be helpful to highlight a few investigations that have focused on treatment issues specifically relevant to SM persons and to consider factors that may influence SM women and men’s access to and benefit from AOD interventions.

Interventions to promote the health of SMs need to address the intersections of multiple minority statuses (e.g., minority sexual orientation, minority race/ethnicity, female gender) and issues such as power, stigma, and victimization (Hatzenbuehler et al. 2013). Positive strategies such as strengthening resilience and promoting family, community, and workplace acceptance have the potential to contribute to long-term health promotion for SM women and men.

Both gender and SM status may affect a person’s ability to find substance abuse treatment that is accessible, affordable, and socially and culturally appropriate. A 2007 review concluded that, although women-only treatment is not necessarily more effective than mixed-gender treatment, treatment approaches that address problems facing substance-abusing women, or that are designed for specific subgroups of women, are more effective (Greenfield et al. 2007).

Along the same lines, SM men and women may benefit from specialized treatment programs especially designed to address the unique issues of SMs, such as coming out; internalized homophobia; violence and discrimination; socialization, dating, and intimacy; family support; and spirituality and religion (Hicks 2000). It may be difficult to find such programs, however, and the lack of available programs may affect choice of and satisfaction with treatment. A telephone survey of substance abuse programs (Cochran et al. 2007*a*) found that 71 percent of agencies with listings indicating sexual minority-specific services did not in fact offer such services. Only 7.4 percent had any kind of specifically tailored treatment.

Using NESARC data to evaluate use of substance abuse treatment among SM adults, McCabe and colleagues (2013) found that, despite having a higher rate of substance use disorders, women who self-identified as lesbian or who reported only same-sex attraction or behavior did not enter substance abuse treatment more often than heterosexual women. The researchers did not find any significant differences in health insurance coverage between lesbian and heterosexual respondents. Likewise, research has found that SM men and women have lower levels of satisfaction with substance abuse treatment compared with heterosexuals (Drabble et al. 2005; Senreich 2009).

In conclusion, although research and clinical interventions are important, broader social and political action is needed to address social determinants of health and to remove barriers to opportunity and equality, whether these barriers are based on gender, minority sexual orientation, age, minority race/ethnicity, low SES, or other marginalized statuses. Such social action may be the ultimate prevention strategy, not only for negative alcohol- related outcomes but also for a wide variety of other health and social problems that affect both SMs and heterosexual persons throughout the world.

## Figures and Tables

**Figure:**
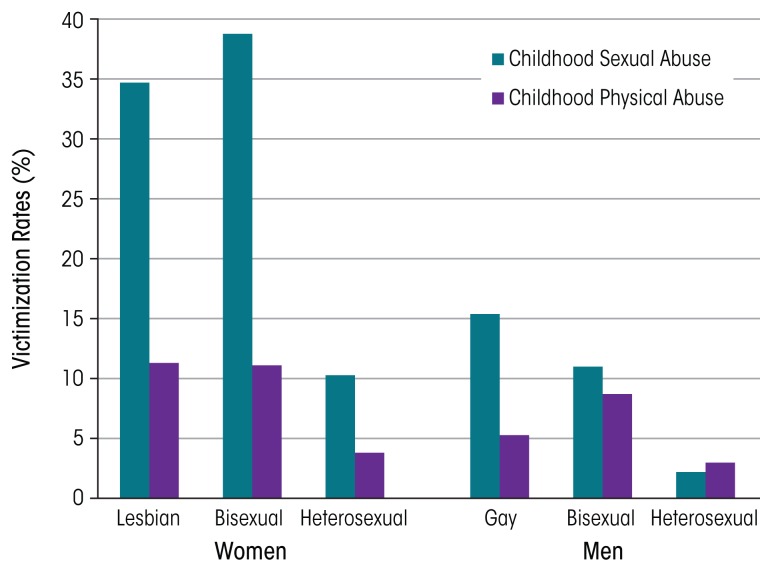
Victimization rates among lesbian/gay, bisexual, and heterosexual women and men, based on findings from the National Epidemiologic Survey on Alcohol and Related Conditions, a nationally representative survey of U.S. adults. SOURCE: Hughes et al. 2010*a*.

## Footnotes

1 The U.S. sample did not include men.

2 There were too few cases of heavy single-occasion alcohol use among lesbians for comparison.

3 Heavy drinking was defined by a gender-specific composite heavy-drinking variable based on five variables that are consistent determinants of alcohol-related health and social problems.

## References

[b1-arcr-38-1-121] Alvy LM, Hughes TL, Kristjanson AF, Wilsnack SC (2013). Sexual identity group differences in child abuse and neglect. Journal of Interpersonal Violence.

[b2-arcr-38-1-121] American Psychiatric Association (APA) (1994). Diagnostic and Statistical Manual of Mental Disorders.

[b3-arcr-38-1-121] Andrews G, Corry J, Slade T, Ezzati M, Rodgers AD, Lopez AD (2004). Child sexual abuse. Comparative Quantification of Health Risks: Global and Regional Burden of Disease due to Selected Major Risk Factors.

[b4-arcr-38-1-121] Austin SB, Jun HJ, Jackson B (2008). Disparities in child abuse victimization in lesbian, bisexual, and heterosexual women in the Nurses’ Health Study II. Journal of Women’s Health.

[b5-arcr-38-1-121] Badgett M, Lee V (2001). Money, Myths, and Change: The Economic Lives of Lesbians and Gay Men..

[b6-arcr-38-1-121] Ball J (2014). More than 2.7 billion people live in countries where being gay is a crime [article online]. The Guardian.

[b7-arcr-38-1-121] Balsam KF, Rothblum ED, Beauchaine TP (2005). Victimization over the life span: A comparison of lesbian, gay, bisexual, and heterosexual siblings. Journal of Consulting and Clinical Psychology.

[b8-arcr-38-1-121] Barnes DM, Hatzenbuehler ML, Hamilton AD, Keyes KM (2014). Sexual orientation disparities in mental health: The moderating role of educational attainment. Social Psychiatry and Psychiatric Epidemiology.

[b9-arcr-38-1-121] Bloomfield K, Grittner U, Kramer S, Gmel G (2006). Social inequalities in alcohol consumption and alcohol-related problems in the study countries of the EU concerted action “Gender, Culture and Alcohol Problems: A Multi-national Study.”. Alcohol and Alcoholism.

[b10-arcr-38-1-121] Bloomfield K, Mäkelä P (2010). Commentary on Huckle et al. (2010): Those confounding facts of lifestyle. Addiction.

[b11-arcr-38-1-121] Bloomfield K, Wicki M, Wilsnack SC (2011). International differences in alcohol use according to sexual orientation. Substance Abuse.

[b12-arcr-38-1-121] Boehmer U (2002). Twenty years of public health research: Inclusion of lesbian, gay, bisexual, and transgender populations. American Journal of Public Health.

[b13-arcr-38-1-121] Brennan PL, Schutte KK, Moos BS, Moos RH (2011). Twenty-year alcohol consumption and drinking problem trajectories of older men and women. Journal of Studies on Alcohol and Drugs.

[b14-arcr-38-1-121] Briere J (1988). The long-term clinical correlates of childhood sexual victimization. Annals of the New York Academy of Sciences.

[b15-arcr-38-1-121] Cochran BN, Peavy KM, Robohm JS (2007a). Do specialized services exist for LGBT individuals seeking treatment for substance misuse? A study of available treatment programs. Substance Use & Misuse.

[b16-arcr-38-1-121] Cochran SD, Grella CE, Mays VM (2012b). Do substance use norms and perceived drug availability mediate sexual orientation differences in patterns of substance use? Results from the California Quality of Life Survey II. Journal of Studies on Alcohol and Drugs.

[b17-arcr-38-1-121] Cochran SD, Mays VM, Alegria M (2007). Mental health and substance use disorders among Latino and Asian American lesbian, gay, and bisexual adults. Journal of Consulting and Clinical Psychology.

[b18-arcr-38-1-121] Collier KL, van Beusekom G, Bos HM, Sandfort TG (2013). Sexual orientation and gender identity/expression related peer victimization in adolescence: A systematic review of associated psychosocial and health outcomes. Journal of Sex Research.

[b19-arcr-38-1-121] Coulter RM, Kenst KS, Bowen DJ (2014). Research funded by the National Institutes of Health on the health of lesbian, gay, bisexual, and transgender populations. American Journal of Public Health.

[b20-arcr-38-1-121] D’Augelli AR, Grossman AH, Starks MT (2008). Gender atypicality and sexual orientation development among lesbian, gay, and bisexual youth: Prevalences, sex differences, and parental responses. Journal of Gay & Lesbian Mental Health.

[b21-arcr-38-1-121] Daulaire N (2014). The importance of LGBT health on a global scale. LGBT Health.

[b22-arcr-38-1-121] Denney JT, Gorman BK, Barrera CB (2013). Families, resources, and adult health: Where do sexual minorities fit?. Journal of Health and Social Behavior.

[b23-arcr-38-1-121] Drabble L, Midanik LT, Trocki K (2005). Reports of alcohol consumption and alcohol-related problems among homosexual, bisexual and heterosexual respondents: Results from the 2000 National Alcohol Survey. Journal of Studies on Alcohol.

[b24-arcr-38-1-121] Drabble L, Trocki KF, Hughes TL (2013). Sexual orientation differences in the relationship between victimization and hazardous drinking among women in the National Alcohol Survey. Psychology of Addictive Behaviors.

[b25-arcr-38-1-121] Dube SR, Anda RF, Felitti VJ (2002). Adverse childhood experiences and personal alcohol abuse as an adult. Addictive Behaviors.

[b26-arcr-38-1-121] Fredriksen-Goldsen KI, Kim HJ, Barkan SE (2013). Health disparities among lesbian, gay, and bisexual older adults: Results from a population-based study. American Journal of Public Health.

[b27-arcr-38-1-121] Gates GJ, Patterson CJ, D’Augelli AR (2012). Demographic perspectives on sexual orientation. Handbook of Psychology and Sexual Orientation.

[b28-arcr-38-1-121] Gates GJ (2013). Same-sex and Different-sex Couples in the American Community Survey: 2005–2011.

[b29-arcr-38-1-121] Greenfield SF, Brooks AJ, Gordon SM (2007). Substance abuse treatment entry, retention, and outcome in women: A review of the literature. Drug and Alcohol Dependence.

[b30-arcr-38-1-121] Hatzenbuehler ML (2009). How does sexual minority stigma “get under the skin”? A psychological mediation framework. Psychological Bulletin.

[b31-arcr-38-1-121] Hatzenbuehler ML, Keyes KM, Hasin DS (2009). State-level policies and psychiatric morbidity in lesbian, gay, and bisexual populations. American Journal of Public Health.

[b32-arcr-38-1-121] Hatzenbuehler ML, McLaughlin KA, Keyes KM, Hasin DS (2010). The impact of institutional discrimination on psychiatric disorders in lesbian, gay, and bisexual populations: A prospective study. American Journal of Public Health.

[b33-arcr-38-1-121] Hatzenbuehler ML, Nolen-Hoeksema S, Erickson SJ (2008). Minority stress predictors of HIV risk behavior, substance use, and depressive symptoms: Results from a prospective study of bereaved gay men. Health Psychology.

[b34-arcr-38-1-121] Hatzenbuehler ML, Phelan JC, Link BG (2013). Stigma as a fundamental cause of population health inequalities. American Journal of Public Health.

[b35-arcr-38-1-121] Herek GM, Gillis JR, Cogan JC (1997). Hate crime victimization among lesbian, gay, and bisexual adults: Prevalence, psychological correlates, and methodological issues. Journal of Interpersonal Violence.

[b36-arcr-38-1-121] Hicks D (2000). The importance of specialized treatment programs for lesbian and gay patients. Journal of Gay & Lesbian Psychotherapy.

[b37-arcr-38-1-121] Holmila M, Raitasalo K (2005). Gender differences in drinking: why do they still exist?. Addiction.

[b38-arcr-38-1-121] Huckle T, You RQ, Casswell S (2010). Socio-economic status predicts drinking patterns but not alcohol-related consequences independently. Addiction.

[b39-arcr-38-1-121] Hughes TL (2005). Alcohol use and alcohol-related problems among lesbians and gay men. Annual Review of Nursing Research.

[b40-arcr-38-1-121] Hughes TL, Johnson TP, Steffen AD (2014). Lifetime victimization, hazardous drinking and depression among heterosexual and sexual minority women. LGBT Health.

[b41-arcr-38-1-121] Hughes T, McCabe SE, Wilsnack SC (2010a). Victimization and substance use disorders in a national sample of heterosexual and sexual minority women and men. Addiction.

[b42-arcr-38-1-121] Hughes T, Szalacha LA, McNair R (2010b). Substance abuse and mental health disparities: Comparisons across sexual identity groups in a national sample of young Australian women. Social Science & Medicine.

[b43-arcr-38-1-121] Hughes TL, Wilsnack SC, Szalacha LA (2006). Age and racial/ethnic differences in drinking and drinking- related problems in a community sample of lesbians. Journal of Studies on Alcohol.

[b44-arcr-38-1-121] Iwamoto DK, Cheng A, Lee CS (2011). “Man-ing” up and getting drunk: The role of masculine norms, alcohol intoxication, and alcohol-related problems among college men. Addictive Behaviors.

[b45-arcr-38-1-121] Kastanis A, Wilson B (2014). Race/Ethnicity, Gender and Socioeconomic Wellbeing of Individuals in Same-sex Couples.

[b46-arcr-38-1-121] Kates J (2014). The U.S. Government and Global LGBT Health: Opportunities and Challenges in the Current Era.

[b47-arcr-38-1-121] Kendler KS, Bulik CM, Silberg J (2000). Childhood sexual abuse and adult psychiatric and substance use disorders in women: An epidemiological and cotwin control analysis. Archives of General Psychiatry.

[b48-arcr-38-1-121] Keyes KM, Li G, Hasin DS (2011). Birth cohort effects and gender differences in alcohol epidemiology: A review and synthesis. Alcoholism: Clinical and Experimental Research.

[b49-arcr-38-1-121] Kim HJ, Fredriksen-Goldsen KI (2012). Hispanic lesbians and bisexual women at heightened risk for health disparities. American Journal of Public Health.

[b50-arcr-38-1-121] Kimmel SB, Mahalik JR (2005). Body image concerns of gay men: The roles of minority stress and conformity to masculine norms. Journal of Consulting and Clinical Psychology.

[b51-arcr-38-1-121] King M, Semlyen J, Tai SS (2008). A systematic review of mental disorder, suicide, and deliberate self-harm in lesbian, gay and bisexual people. BMC Psychiatry.

[b52-arcr-38-1-121] Krivickas KM (2010). Same-sex Couple Households in the U.S., 2009.

[b53-arcr-38-1-121] Kuang MF, Mathy RM, Carol HM (2004). The effects of sexual orientation, gender identity, and gender role on the mental health of women in Taiwan’s T-Po lesbian community. Journal of Psychology and Human Behavior.

[b54-arcr-38-1-121] Kuntsche S, Knibbe RA, Gmel G (2009). Social roles and alcohol consumption: A study of 10 industrialised countries. Social Science & Medicine.

[b55-arcr-38-1-121] Kuntsche S, Knibbe RA, Kuntsche E, Gmel G (2011). Housewife or working mum—each to her own? The relevance of societal factors in the association between social roles and alcohol use among mothers in 16 industrialized countries. Addiction.

[b56-arcr-38-1-121] Lewis RJ, Derlega VJ, Berndt A (2001). An empirical analysis of stressors for gay men and lesbians. Journal of Homosexuality.

[b57-arcr-38-1-121] Lewis RJ, Derlega VJ, Griffin JL (2003). Stressors for gay men and lesbians: Life stress, gay-related stress, stigma consciousness, and depressive symptoms. Journal of Social and Clinical Psychology.

[b58-arcr-38-1-121] Lippa RA (2000). Gender-related traits in gay men, lesbian women, and heterosexual men and women: The virtual identity of homosexual-heterosexual diagnosticity and gender diagnosticity. Journal of Personality.

[b59-arcr-38-1-121] Liu H, Reczek C, Brown D (2013). Same-sex cohabitors and health: The role of race-ethnicity, gender, and socioeconomic status. Journal of Health and Social Behavior.

[b60-arcr-38-1-121] Locatelli D, Sanchez Z, Opaleye E (2012). Socioeconomic influences on alcohol use patterns among private school students in São Paulo. Revista Brasileira de Psiquiatria.

[b61-arcr-38-1-121] Mäkelä P, Tigerstedt C, Mustonen H (2012). The Finnish drinking culture: Change and continuity in the past 40 years. Drug and Alcohol Review.

[b62-arcr-38-1-121] Mayock P, Bryan A, Carr N (2008). Supporting LGBT lives: A Study of the Mental Health and Well-being of Lesbian, Gay, Bisexual and Transgender People.

[b63-arcr-38-1-121] Mays VM, Cochran SD (2001). Mental health correlates of perceived discrimination among lesbian, gay, and bisexual adults in the United States. American Journal of Public Health.

[b64-arcr-38-1-121] McCabe SE, Bostwick WB, Hughes TL (2010). The relationship between discrimination and substance use disorders among lesbian, gay, and bisexual adults in the United States. American Journal of Public Health.

[b65-arcr-38-1-121] McCabe SE, Hughes TL, Bostwick WB (2009). Sexual orientation, substance use behaviors and substance dependence in the United States. Addiction.

[b66-arcr-38-1-121] McCabe SE, West BT, Hughes TL, Boyd CJ (2013). Sexual orientation and substance abuse treatment utilization in the United States: Results from a national survey. Journal of Substance Abuse Treatment.

[b67-arcr-38-1-121] McKirnan DJ, Peterson PL (1989a). Alcohol and drug use among homosexual men and women: Epidemiology and population characteristics. Addictive Behaviors.

[b68-arcr-38-1-121] McKirnan DJ, Peterson PL (1989b). Psychosocial and cultural factors in alcohol and drug abuse: An analysis of a homosexual community. Addictive Behaviors.

[b69-arcr-38-1-121] Melotti R, Lewis G, Hickman M (2013). Early life socio-economic position and later alcohol use: Birth cohort study. Addiction.

[b70-arcr-38-1-121] Mereish EH, Bradford JB (2014). Intersecting identities and substance use problems: Sexual orientation, gender, race, and lifetime substance use problems. Journal of Studies on Alcohol and Drugs.

[b71-arcr-38-1-121] Meyer IH (1995). Minority stress and mental health in gay men. Journal of Health and Social Behavior.

[b72-arcr-38-1-121] Meyer IH (2003). Prejudice, social stress, and mental health in lesbian, gay, and bisexual populations: Conceptual issues and research evidence. Psychological Bulletin.

[b73-arcr-38-1-121] Nelson EC, Heath AC, Madden PA (2002). Association between self-reported childhood sexual abuse and adverse psychosocial outcomes: Results from a twin study. Archives of General Psychiatry.

[b74-arcr-38-1-121] Nguyen N, Walters ST, Wyatt TM, DeJong W (2011). Use and correlates of protective drinking behaviors during the transition to college: Analysis of a national sample. Addictive Behaviors.

[b75-arcr-38-1-121] Ortiz-Hernandez L, Tello BL, Valdes J (2009). The association of sexual orientation with self-rated health, and cigarette and alcohol use in Mexican adolescents and youths. Social Science & Medicine.

[b76-arcr-38-1-121] Pan American Health Organization (PAHO) (2012). Addressing the Causes of Disparities in Health Service Access and Utilization for Lesbian, Gay, Bisexual and Trans (LGBT) Persons.

[b77-arcr-38-1-121] Pan American Health Organization (PAHO) (2013). Health Authorities Pledge to Improve Access to Health Care for LGBT People.

[b78-arcr-38-1-121] Pew Research Center (2015). Gay Marriage Around the World.

[b79-arcr-38-1-121] Pitel L, Madarasova Geckova A, Reijneveld SA, van Dijk JP (2013). Socioeconomic gradient shifts in health-related behaviour among Slovak adolescents between 1998 and 2006. International Journal of Public Health.

[b80-arcr-38-1-121] Ryan C, Huebner D, Diaz RM, Sanchez J (2009). Family rejection as a predictor of negative health outcomes in White and Latino lesbian, gay, and bisexual young adults. Pediatrics.

[b81-arcr-38-1-121] Sandmaier M (1980). The Invisible Alcoholics: Women and Alcohol Abuse in America.

[b82-arcr-38-1-121] Senreich E (2009). A comparison of perceptions, reported abstinence, and completion rates of gay, lesbian, bisexual, and heterosexual clients in substance abuse treatment. Journal of Gay & Lesbian Mental Health.

[b83-arcr-38-1-121] Substance Abuse and Mental Health Services Administration (SAMHSA) (2013). Results from the 2012 National Survey on Drug Use and Health: Summary of National Findings.

[b84-arcr-38-1-121] Szymanski DM, Chung YB (2001). The Lesbian Internalized Homophobia Scale: A rational/theoretical approach. Journal of Homosexuality.

[b85-arcr-38-1-121] Szymanski DM, Chung YB, Balsam KF (2001). Psychosocial correlates of internalized homophobia in lesbians. Measurement and Evaluation in Counseling and Development.

[b86-arcr-38-1-121] Talley AE, Hughes TL, Aranda F (2014). Exploring alcohol-use behaviors among heterosexual and sexual minority adolescents: Intersections with sex, age, and race/ethnicity. American Journal of Public Health.

[b87-arcr-38-1-121] Tjaden P, Thoennes N, Allison CJ (1999). Comparing violence over the life span in samples of same-sex and opposite-sex cohabitants. Violence and Victims.

[b88-arcr-38-1-121] Trocki KF, Drabble L, Midanik L (2005). Use of heavier drinking contexts among heterosexuals, homosexuals and bisexuals: Results from a national household probability survey. Journal of Studies on Alcohol.

[b89-arcr-38-1-121] Vaeth PA, Caetano R, Rodriguez LA (2012). The Hispanic Americans Baseline Alcohol Survey (HABLAS): The association between acculturation, birthplace and alcohol consumption across Hispanic national groups. Addictive Behaviors.

[b90-arcr-38-1-121] van Griensven F, Kilmarx PH, Jeeyapant S (2004). The prevalence of bisexual and homosexual orientation and related health risks among adolescents in northern Thailand. Archives of Sexual Behavior.

[b91-arcr-38-1-121] van Oers JA, Bongers IM, van de Goor LA, Garretsen HF (1999). Alcohol consumption, alcohol-related problems, problem drinking, and socioeconomic status. Alcohol and Alcoholism.

[b92-arcr-38-1-121] Wilsnack RW, Vogeltanz ND, Wilsnack SC (2000). Gender differences in alcohol consumption and adverse drinking consequences: Cross-cultural patterns. Addiction.

[b93-arcr-38-1-121] Wilsnack RW, Wilsnack SC, Boyle P, Boffetta P, Lowenfel AB (2013). Gender and alcohol: Consumption and consequences. Alcohol: Science, Policy, and Public Health.

[b94-arcr-38-1-121] Wilsnack RW, Wilsnack SC, Kristjanson AF (2009). Gender and alcohol consumption: Patterns from the multinational GENACIS project. Addiction.

[b95-arcr-38-1-121] Wilsnack RW, Wilsnack SC, Obot IS, Obot IS, Room R (2005). Why study gender, alcohol, and culture?. Alcohol, Gender and Drinking Problems: Perspectives from Low and Middle Income Countries.

[b96-arcr-38-1-121] Wilsnack SC, Hughes TL, Johnson TP (2008). Drinking and drinking-related problems among heterosexual and sexual minority women. Journal of Studies on Alcohol and Drugs.

[b97-arcr-38-1-121] Wilsnack SC, Kristjanson AF, Hughes TL, Benson PW (2012). Characteristics of childhood sexual abuse in lesbians and heterosexual women. Child Abuse & Neglect.

[b98-arcr-38-1-121] Wilsnack SC, Wilsnack RW, Kristjanson AF, Koenig LJ, Doll LS, O’Leary A (2004). Child sexual abuse and alcohol use among women: Setting the stage for risky sexual behavior. From Child Sexual Abuse to Adult Sexual Risk: Trauma, Revictimization, and Intervention.

[b99-arcr-38-1-121] Witbrodt J, Mulia N, Zemore SE, Kerr WC (2014). Racial/ethnic disparities in alcohol-related problems: Differences by gender and level of heavy drinking. Alcoholism: Clinical and Experimental Research.

[b100-arcr-38-1-121] World Health Organization (WHO) (2014). Global Status Report on Alcohol and Health.

